# Unprecedented hour-long residence time of a cation in a left-handed G-quadruplex†

**DOI:** 10.1039/d1sc00515d

**Published:** 2021-04-26

**Authors:** Fernaldo Richtia Winnerdy, Blaž Bakalar, Poulomi Das, Brahim Heddi, Adrien Marchand, Frédéric Rosu, Valérie Gabelica, Anh Tuân Phan

**Affiliations:** School of Physical and Mathematical Sciences, Nanyang Technological University Singapore 637371 Singapore phantuan@ntu.edu.sg; Laboratoire de Biologie et de Pharmacologie Appliquée, CNRS, Ecole Normale Supérieure Paris-Saclay Gif-sur-Yvette 91190 France; Laboratoire Acides Nucléiques: Régulations Naturelle et Artificielle, Université de Bordeaux, Inserm & CNRS (ARNA, U1212, UMR5320), IECB Pessac 33600 France; Institut Européen de Chimie et Biologie, Université de Bordeaux, CNRS & Inserm (IECB, UMS3033, US001) Pessac 33607 France

## Abstract

Cations are critical for the folding and assembly of nucleic acids. In G-quadruplex structures, cations can bind between stacked G-tetrads and coordinate with negatively charged guanine carbonyl oxygens. They usually exchange between binding sites and with the bulk in solution with time constants ranging from sub-millisecond to seconds. Here we report the first observation of extremely long-lived K^+^ and NH_4_^+^ ions, with an exchange time constant on the order of an hour, when coordinated at the center of a left-handed G-quadruplex DNA. A single-base mutation, that switched one half of the structure from left- to right-handed conformation resulting in a right–left hybrid G-quadruplex, was shown to remove this long-lived behaviour of the central cation.

## Abstract 2

An extremely long-lived cation has been detected in left-handed G-quadruplexes.

## Graphical abstract

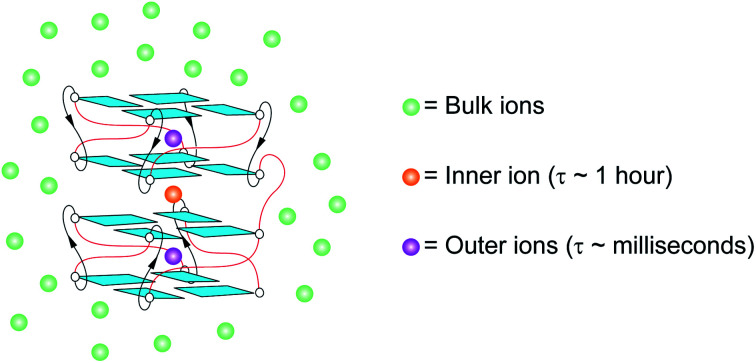

## Introduction

The timescales of the dynamics of biomolecular systems play a crucial role in the behavior of the participating molecules and are linked to their biological functions.^1,2^ For instance, water molecules flip their hydrogen bonding in bulk on the order of picoseconds,^3,4^ while the residence time of water molecules in the hydration shells of biomolecules is in the order of nanoseconds.^5,6^ The reduced dynamics of water molecules in the hydration shells has a major impact on the folding, structural, kinetic and thermodynamic properties of encased biomolecules.^5,7–9^ Similarly, the efficacy of ligands and effectiveness of drugs are linked to their residence time, upon binding to their targets^10,11^ with values ranging from seconds,^12^ hours^13^ to days.^14^

Cations are considered an integral part of DNA structures. They interact with and shield the negative charges of the phosphate backbone,^15^ thus aiding the condensation of DNA structures.^16^ The residence time of sodium ions around the phosphate backbone was found to be on the nanosecond timescale based on ^23^Na nuclear relaxation experiments as well as molecular dynamics simulations.^17,18^ G-quadruplex structures are formed by stacking of square-planar structures, termed G-tetrads, and are highly polymorphic.^19–22^ Cations have been found within G-quadruplex structures, where they coordinate with the carbonyl oxygens and help stabilize the G-tetrad core.^23,24^ While some G-quadruplexes do not undergo significant structural changes in various cation solutions,^25^ structures such as human telomeric G-quadruplexes are particularly sensitive to the cations present in solution.^26–36^ Cations are either coordinated between stacked G-tetrads or in the plane of a G-tetrad, depending on their respective ionic radius and charge.^20,37–39^ Previous studies using ^15^NH_4_^+^ ions have shown that they dynamically exchange between binding sites as well as with the bulk ions present in solution.^38,40–45^ The residence times determined for the coordinated cations span several orders of magnitude, from sub-millisecond,^41,42,46,47^ millisecond,^38,40^ to 1.7 seconds, the longest time observed so far.^44^

The first left-handed G-quadruplex structure (named *Z-G4*) has been reported for a natural DNA sequence.^48^ The left-handedness of *Z-G4* does not pertain to its stereochemistry, but rather to the base stacking and phosphate backbone progression. While investigating the effects and behaviors of cations in the context of left-handed G-quadruplexes, we unexpectedly found extremely long-lived cations, K^+^ and/or NH_4_^+^, residing inside the channel of the *Z-G4* structure, as detected by NMR spectroscopy and mass spectrometry. The idea of G-quadruplexes as targets for therapeutics was described before.^49–51^ The *Z-G4* structure was shown to be specifically recognized by a chiral ligand.^52^ This work implies a steadfast *Z-G4* conformation with respect to time, which may be important for its potential biological functions as an aptamer or drug target. The combination of unique structural features of *Z-G4* and long-lasting cations in the channel can be useful in G-quadruplex engineering and nanotechnology.^53,54^

## Results and discussion

### *Z-G4* is formed in K^+^ and NH_4_^+^ solution

**Fig. 1:**
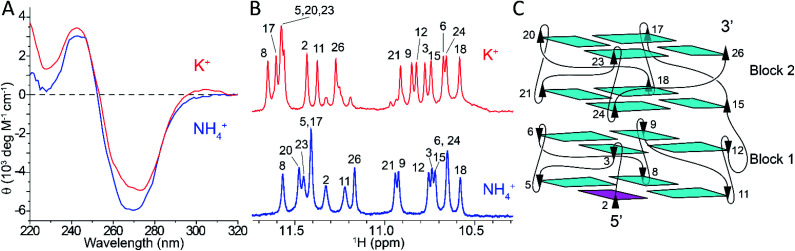
(A) CD spectra of *Z-G4* in K^+^ (red) or NH_4_^+^ (blue). (B) Imino proton spectra of *Z-G4* in either of the ions with assignments indicated by residue numbers. (C) Schematic representation of *Z-G4* folded in either K^+^ or NH_4_^+^.

The folding of *Z-G4* (Table S1†) was tested in the presence of different monovalent cations. We observed the formation of G-quadruplex structures with left-handed CD and NMR spectral characteristics in K^+^ and NH_4_^+^ solution, but not in Cs^+^, Na^+^, and Li^+^ likely due to their differing ionic radii (Fig. S1†). The CD spectra of *Z-G4* in K^+^ and NH_4_^+^ solutions (Fig. 1A) had a dip at 270 nm and a peak at 245 nm, consistent with previous observations for left-handed G-quadruplexes.^48,55^ The NMR spectra of *Z-G4* in K^+^ and NH_4_^+^ (Fig. 1B) indicated the formation of a single major G-quadruplex structure with a similar distribution of the 16 imino protons peaks. A particular structural feature of the left-handed G-quadruplex is the formation of hydrogen bonds between O4′ of the capping thymines and guanine amino protons of the adjacent G-tetrad,^48,55^ resulting in the observation of amino protons at ∼9 ppm in K^+^ and NH_4_^+^ solution (Fig. S2†).^56^

Spectral assignments of *Z-G4* in NH_4_^+^ solution were obtained using both site-specific labeling method^57^ and NOESY spectral analysis (Fig. S3†). NMR spectral analysis indicated that the same G-quadruplex fold (Fig. 1C)—two bi-layered left-handed G-quadruplex blocks (*Block1* and *Block2*)—was preserved in both K^+^ and NH_4_^+^ solutions. This conclusion is also supported by D_2_O solvent exchange experiments, where only imino protons peaks of inner G-tetrads were preserved (Fig. S2†).

### Observation of coordinated ammonium ions in *Z-G4* by NMR

The folding of *Z-G4* in ^15^NH_4_^+^ solution produced a new set of proton peaks around 6.5–7.0 ppm compared to the K^+^ counterpart. The additional peaks were also observed in a ^15^N-filtered experiment and therefore assigned to the protons of ammonium ions (Fig. S4†). At pH 7, we observed peaks at 6.87 and 6.57 ppm, with an area ratio of approximately 2 : 1 (Fig. S4C†). These peaks were assigned to three ammonium ions coordinated in the G-tetrad core of *Z-G4*. Decreasing the pH to 5 slowed the proton exchange rate and revealed the bulk ammonium peak at 7.02 ppm (Fig. S5†).

**Fig. 2:**
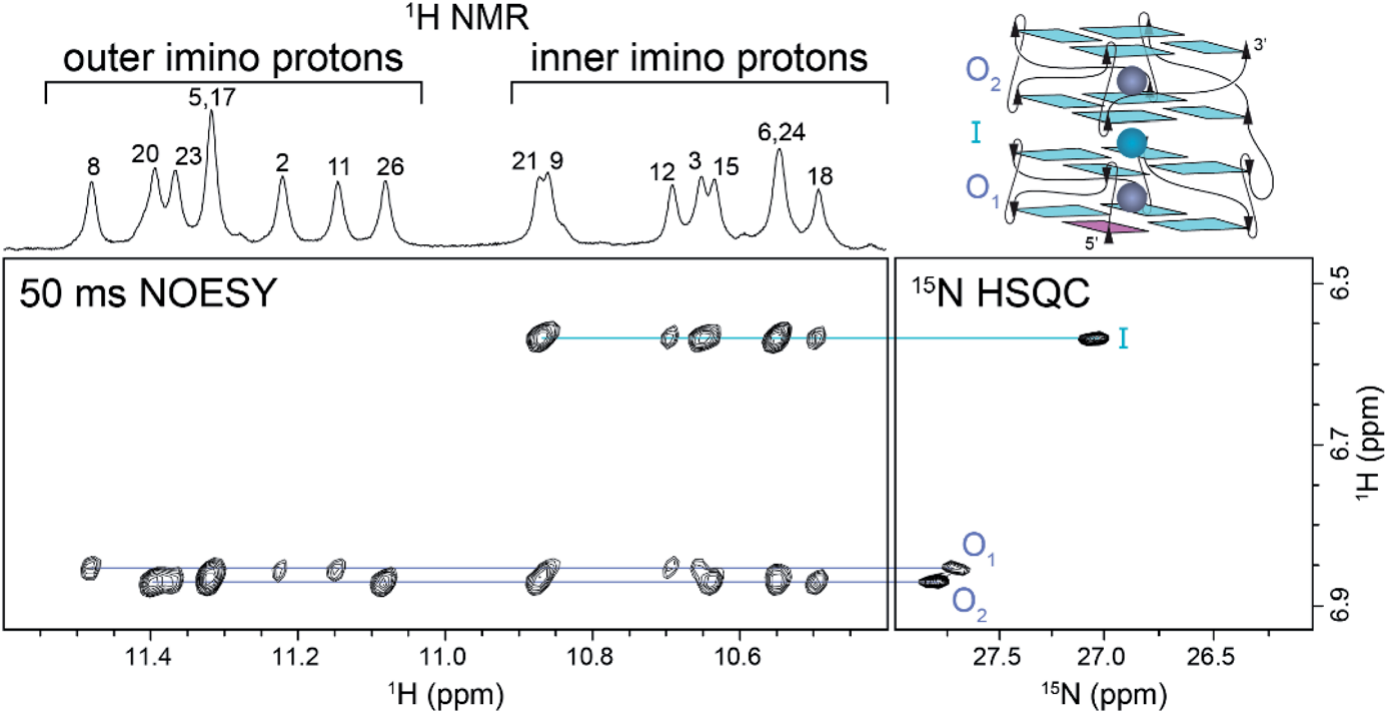
NOESY and ^15^N–^1^H HSQC experiments at 283 K correlating the ^1^H chemical shifts with the position of the ammonium ions within the *Z-G4* structure. Outer and inner imino proton peaks are labeled in the NOESY spectrum. The outer ions are shown in purple, while the inner ion is shown in cyan.

We correlated the coordination positions of the ammonium ions with the neighboring protons by using NOESY and ^15^N–^1^H HSQC experiments (Fig. 2). The upfield ammonium ion peak at 6.57 ppm was correlated with a single ^15^N–^1^H HSQC cross-peak and had NOE cross-peaks only with the inner G-tetrad imino protons (Fig. 2, cyan line). We concluded that this ammonium ion occupied the inner binding site, between the two blocks of *Z-G4* (Fig. 2, cyan sphere). The downfield ammonium ion peak at 6.87 ppm was resolved in 2D experiments, giving rise to two discrete cross-peaks in the ^15^N–^1^H HSQC spectrum. Each proton showed eight distinct NOE cross-peaks, four with outer tetrad imino protons and four with inner tetrad imino protons (Fig. 2, purple lines). These results indicated that both downfield ammonium ion peaks are coordinated within the two outer binding sites of the *Z-G4* structure (Fig. 2, purple spheres). Based on the imino proton assignments, we determined that O_1_ corresponds to the ion in *Block1* and O_2_ to the ion in *Block2*.

A 2D NzEx-HSQC experiment^38^ with 100 ms mixing time was performed for *Z-G4* folded in ^15^NH_4_^+^ at pH 5.1 and 293 K. The introduction of a mixing time allowed the exchange process between the ^15^NH_4_^+^ ions in the binding sites and in solution to be observed *via* correlation cross-peaks in the NMR spectrum. We observed that the outer ions produced cross-peaks with the bulk ^15^NH_4_^+^ (Fig. S6†). On the other hand, the inner ion did not produce any cross-peaks, with either the outer ions or the bulk ^15^NH_4_^+^, indicating that its exchange time was much longer than the mixing time.^41^ Further variations of the mixing time and temperature provided us with a rough estimate of the outer ion exchange time with bulk ammonium to be on the order of 100 ms.

### Tracking ^15^NH_4_^+^ and K^+^ ion exchange using ^1^H NMR

**Fig. 3:**
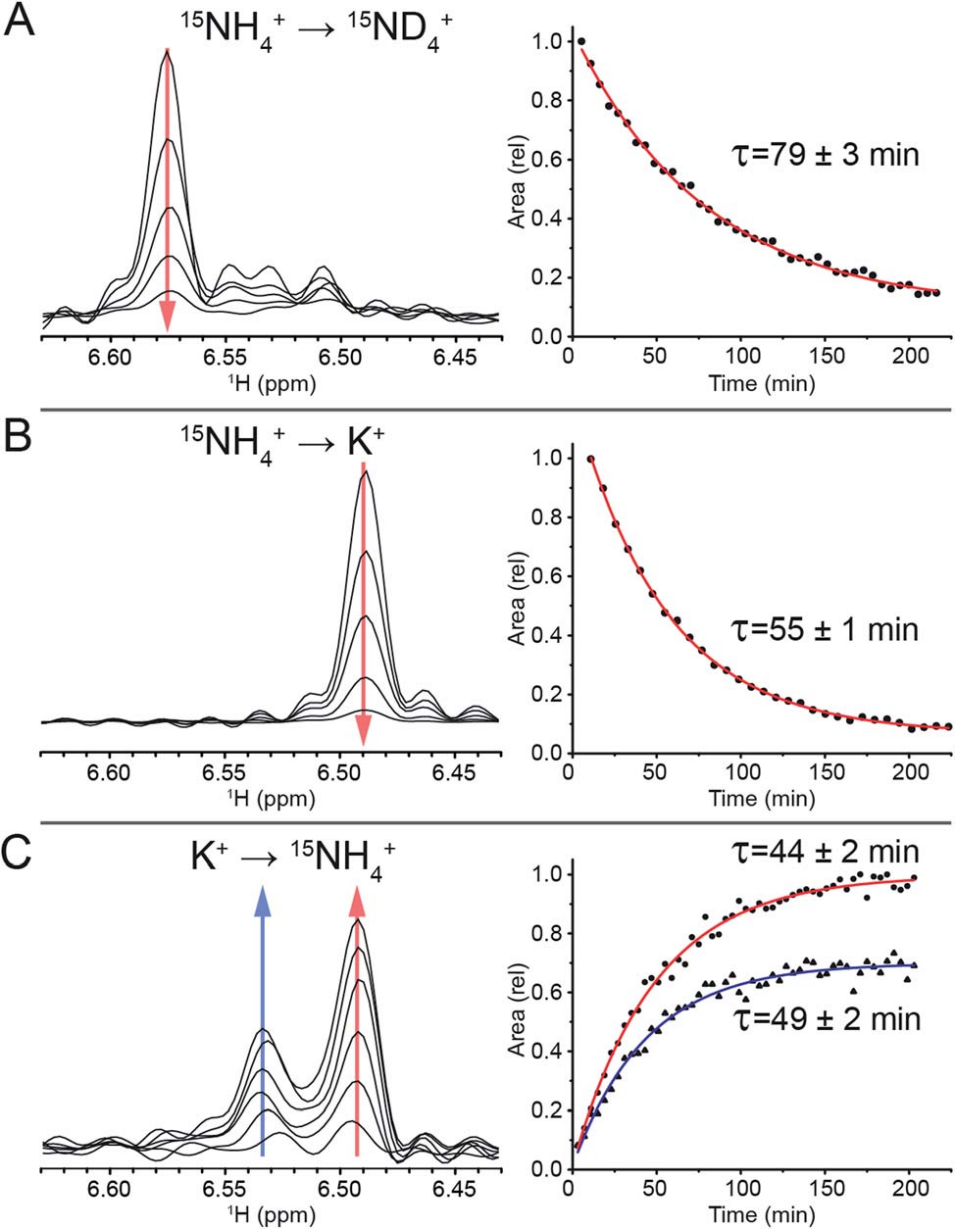
Tracking of the inner ammonium peak (dis)appearance with ^15^N-filtered ^1^H NMR. (A) ^15^NH_4_^+^ peak disappearance as it is exchanged with the bulk ^15^ND_4_^+^. (B) ^15^NH_4_^+^ peak disappearance as it is exchanged with the bulk K^+^. (C) ^15^NH_4_^+^ peak appearance as it replaces the coordinated K^+^ ion.

To track the NH_4_^+^ ion exchange, we first performed a D_2_O exchange experiment at 298 K and pH 7. In this experiment, we observed that the outer ammonium ion proton peaks disappeared immediately, while the inner ammonium ion peak remained observable for a prolonged period of time. The area of the latter peak decreased exponentially, with a time constant of *τ* = (79 ± 3) min (Fig. 3A). This exchange time is three orders of magnitude longer than previous longest residence times reported for ions coordinated within G-quadruplex structures.^44^

To further study this novel behavior of the inner ion, we investigated the exchange between NH_4_^+^ and K^+^ ions. Titration of K^+^ in a sample containing 100 mM NH_4_^+^ revealed three states of the inner ammonium ion, with outer ions being either 2 NH_4_^+^, 1 NH_4_^+^ + 1 K^+^, or 2 K^+^, corresponding to proton peaks at 6.57, 6.53 and 6.49 ppm respectively (Fig. S7†). Note that in the presence of both 100 mM NH_4_^+^ and 100 mM K^+^, the species containing 2 K^+^ at the outer binding sites predominates, indicating a higher affinity of K^+^ to these sites.

A sample of *Z-G4* was folded in 100 mM ^15^NH_4_^+^ and subsequently dialyzed against H_2_O for 10 minutes in order to remove most of the bulk ^15^NH_4_^+^ ions in solution. It was flash frozen and lyophilized. In dried condition, the sample was re-suspended in a buffer containing 100 mM K^+^ ions and immediately tracked with time-series ^15^N-filtered ^1^H NMR experiments. The outer ^15^NH_4_^+^ ions were quickly replaced by K^+^ ions. We observed the area of the inner ^15^NH_4_^+^ peak disappearing with a time constant of *τ* = (55 ± 1) min (Fig. 3B), which can be attributed to the exchange process of the inner ^15^NH_4_^+^ with K^+^. Concurrently, the imino proton spectra showed the disappearance of the initial species (NH_4_^+^ form) and the appearance of a new species (K^+^ form) (Fig. S8†). The time constants for the appearance and disappearance of the corresponding imino protons were found to be consistent with that of the decreasing inner ammonium ion peak. These values directly correspond to the residence time of the inner ^15^NH_4_^+^ ion in *Z-G4*. Any reassociation process of ^15^NH_4_^+^ ion was ruled out due to the huge abundance of bulk K^+^ counterions, leaving the concentration of released ^15^NH_4_^+^ ions negligible. As a control experiment, ^15^NH_4_^+^ to K^+^ ion exchange was also performed with the sequence TG_4_T,^58^ which forms a tetrameric right-handed G-quadruplex. The results displayed fast exchange of channel ions, further highlighting the uniqueness of the ion with long residence time in *Z-G4* (Fig. S9†). This control experiment is also supported by previous ion-exchange experiments on various right-handed G-quadruplexes.^41,42,59^

To determine the specificity of the unprecedented slow exchange rates towards the type of the ion, we conducted the experiment in reverse. We prepared a sample of *Z-G4* folded in K^+^ then re-suspended it in 100 mM ^15^NH_4_^+^. Over the course of 3 hours, we observed the emergence of two inner ammonium ion peaks (Fig. 3C) corresponding to different configurations of the outer ion occupancies. The peak at 6.53 ppm corresponded to *Z-G4* with one of each ion type (^15^NH_4_^+^ and K^+^) as outer ions, while the peak at 6.49 ppm corresponded to *Z-G4* with K^+^ as both outer ions (Fig. S7†). By tracking the area of both inner ion peaks over time (Fig. 3C) we observed time constants of *τ* = (49 ± 2) min and *τ* = (44 ± 2) min respectively. These time constants are slightly shorter than those observed in the previous experiments, where K^+^ was replacing ^15^NH_4_^+^. In addition, NMR spectrum of *Z-G4* at time *t* = 210 min after resuspension in ^15^NH_4_^+^ solution indicated the presence of multiple conformations (Fig. S10†), corresponding to different combinations of bound ions as observed in ^15^N-filtered experiments (not shown). This observation is consistent with NH_4_^+^ ions having lower affinity to the *Z-G4* binding sites compared to K^+^: in similar experiments K^+^ can almost fully replace NH_4_^+^, while NH_4_^+^ can only partially replace K^+^ at the binding sites of *Z-G4*. To further support this claim, we performed non-competition-based control experiments, whereby we re-suspended the *Z-G4* samples prepared in K^+^ or ^15^NH_4_^+^ with deionized H_2_O (Fig. S11†). The results supported the long-lived properties of both ions in *Z-G4*.

The different estimated values of the NH_4_^+^ time constants in the two methods discussed above could be partly attributed to the different ions that were present in each experiment. Previously, adding Na^+^ to NH_4_^+^-containing solvent was shown to accelerate the movements of the NH_4_^+^ inside the G-quadruplex channel.^59^

To probe the structural impact on the long residence time of the inner channel ions, mutation studies of *Z-G4* were performed. Mutations that preserve the left-handed G4 structure, such as the sequence *2xBlock2Δ*,^55^ were found to possess similar property of long-lived inner ions (Fig. S12 and S13†). Another mutated sequences, *ZG4-T4mod*, which has a right–left hybrid G4 conformation (Fig. S14†),^60^ was unable to fold in NH_4_^+^ (Fig. S15†) and thus the K^+^ to NH_4_^+^ ion exchange experiment could not be performed. Hence, we opted to perform mass spectrometry experiments to detect the exchange between the channel ^41^K^+^ and bulk ^39^K^+^ isotopes (Fig. S16†). The results are further described in the mass spectrometry section.

### Observation and tracking of ions in the channel of *Z-G4* using mass spectrometry

Mass spectrometry (MS) was used to track the ion exchange in the intact *Z-G4* (using non-destructive ionization and desolvation conditions) by exploiting the different masses of NH_4_^+^ and K^+^ ions (18 and 39 Da respectively). A folded G-quadruplex molecule with coordinated K^+^ or NH_4_^+^ ions can be detected at a particular *m*/*z* ratio depending on the number of each ion coordinated within the G-quadruplex.

**Fig. 4:**
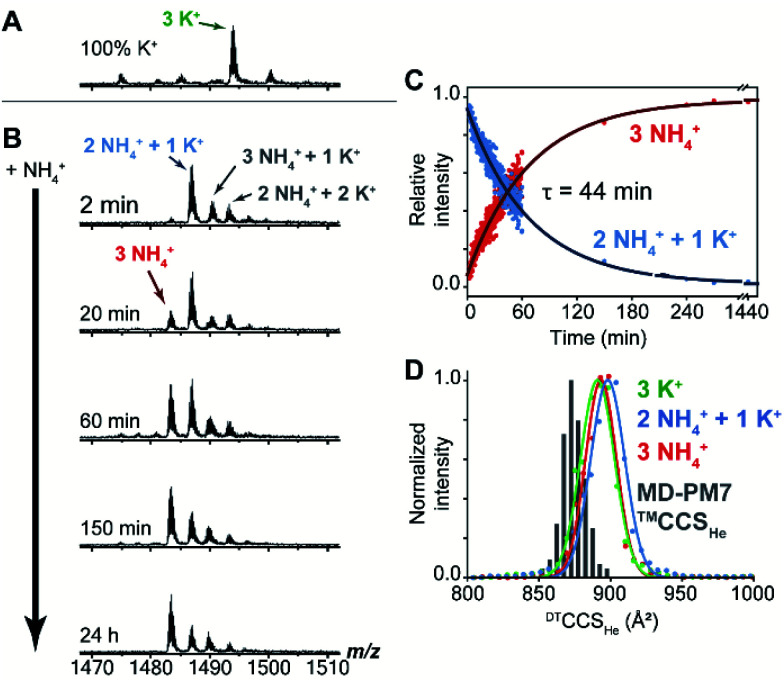
(A) Native negative-ion electrospray ionization-mass spectrometry data for *Z-G4* folded in K^+^, (B) subsequently diluted with NH_4_^+^ buffer and tracked with time (zooms on the 6-charge state). The 3 NH_4_^+^ species is annotated in red and the 2 NH_4_^+^ + 1 K^+^ in blue. Time of spectrum collection is shown on the left. Additional observed species are marked in black (3 NH_4_^+^ + 1 K^+^ and 2 NH_4_^+^ + 2 K^+^), presumably correspond to one extra non-specific adduct. (C) Evolution of the relative intensities of the two main species (3 NH_4_^+^ and 2 NH_4_^+^ + 1 K^+^) as a function of time. The contribution of the non-specific adducts has been taken into account following a previously published method. (D) Histogram of the calculated collision cross section (CCS) values compared with the distributions experimentally obtained by drift tube ion mobility.

A sample of *Z-G4* was prepared in 10 mM KCl and 90 mM trimethylammonium acetate, heated to 90 °C, slowly cooled to room temperature, and stored for 7 days. A control mass spectrum was recorded by diluting the stock sample to 1 mM KCl and 99 mM TMAA. We detected three specifically bound K^+^ ions (Fig. 4A). Then, the same stock sample was diluted with ammonium acetate, to final concentrations of 1 mM KCl, 9 mM TMAA and 90 mM NH_4_OAc, and the spectra were recorded at several time points (Fig. 4B). Within the dead time of the experiment (2 min), two K^+^ ions were replaced by NH_4_^+^ ions. A few extra adducts were observed (total of four cations bound), and these were presumably nonspecific adducts to outer sites (NH_4_OAc is less good than TMAA to suppress nonspecific adducts).^61^ We monitored the slow disappearance of the complex with 1 K^+^ and 2 NH_4_^+^ (blue arrow) and the appearance of the complex with 3 NH_4_^+^ (red arrow). The distribution of nonspecific adducts was estimated from the time point at 24 h, and this contribution was mathematically subtracted to construct the decay curves (Fig. 4C). The slow emergence of the 3 NH_4_^+^ species (*τ* = 44 min) agrees with our NMR observations where we also observed the fast exchange of the outer ions followed by an extremely slow exchange of the inner ion.

We also checked the gas-phase ion mobilities of the complexes with 3 K^+^, 1 K^+^ + 2 NH_4_^+^ and 3 NH_4_^+^. The arrival time distributions for the ion mobility separation in helium, here converted to collision cross section (CCS) distributions for comparison with theoretical values, were superimposable and relatively narrow, indicating no structural change upon cation replacement. The histogram of calculated CCS values overlaps very well with the experimental distributions (Table S3† and Fig. 4D). The gas-phase simulated structures, obtained by PM7 semi-empirical molecular dynamics, differ from the X-ray crystal structure mainly by: (1) the thymine loop rearrangements and (2) the narrower grooves due to hydrogen bond formation between the partially neutralized phosphate groups. The latter phenomenon, previously described for B-DNA duplexes,^62^ does not significantly perturb the G-tetrad core of *Z-G4* (Fig. S17†).

Mass spectrometry experiments were also performed to compare the K^+^ → K^+^ ion exchange process in *Z-G4* (left-handed) and its single-base mutated sequence *ZG4-T4mod* (right–left hybrid). The molecules were initially folded in presence of ^41^K^+^ isotopes then were diluted in ^39^K^+^ solvent. The results showed that the exchange times for the two outer ions for both constructs were fast, while the inner ions stayed longer for the *Z-G4* construct (Fig. S16†). By 25 minutes, almost all the ^41^K^+^ inner ions in *Z-G4* were still present, while around 50% of the ^41^K^+^ inner ions in *ZG4-T4mod* were already exchanged out. After 3 days, the *Z-G4* construct still contained most of its inner ^41^K^+^ ions, while *ZG4-T4mod* construct lost all the inner ^41^K^+^ ions. Due to different experimental conditions, the approximation of the residence time of the central cation in this experiment is not to be compared with the NMR results for exchange between K^+^ and NH_4_^+^. However, in relative terms, we observed that the residence time of cations residing in the pure left-handed structure (*Z-G4*) was much longer compared to the ones in the right-left hybrid structure (*ZG4-T4mod*). These data show that certain structural features the left-handed G-quadruplex is required for the long residence time of the central ion, as a single-base mutation which affected the left-handed conformation could alter the residence time.

### Long-lived cations: possible cause and applications

It was shown previously that the rate of ion exchange between the G-quadruplex channel and the bulk is not proportional to the rate of G-tetrad base opening, suggesting that the G-quadruplex structure does not need to be opened in order for the ions to migrate.^37,63^ Several factors that affect the ion residence time inside the G-quadruplex channel were identified, such as the molecularity,^64^ size of cations,^38,65^ steric hindrances,^42^ guanine conformations and strand directionality.^66^ Based on these studies, we presumed that the ions inside a left-handed G-quadruplex structure, especially the inner ions, bind tighter to their individual binding sites. The behavior might not necessarily be directly due to the left-handedness of *Z-G4*, however, the unique twist on the phosphate backbone and the different relative conformations between the guanine bases and the sugars resulted from the left-handedness, including the hydrogen bonds formed between the O4′ sugars of thymine loops and the amino groups of neighboring guanines, might inhibit the G-tetrad breathing dynamics, and therefore the ion movements. Further experimental or computational studies are required to fully explain the phenomenon, as there has been no preceding analytical research on hour-long-lived cations in nucleic acid structures.

In general, G4 conformations are highly dependent on the presence and type of cations. Crowding conditions and the presence of counterions can affect the stability of G4 structures, with some divalent ions being able to even disrupt the G4 structures entirely.^67–71^ Therefore, the long-lasting property of the ions in the *Z-G4* could potentially be useful in nanotechnology. For example, G-tetrads could be used as the basis for construction of artificial nanopores/ion channels^72^ and the ability to control the ion residence times might find useful applications.

## Conclusions

We report an unprecedented hour-long residence time of the central ion in the core of the left-handed G-quadruplex *Z-G4*. We showed that this long-lived property of the central ion was removed upon a single-base mutation that disrupted the left-handed conformation. These results highlight the key role of the central cation and the interface between the two subunits in forming the left-handed G-quadruplex conformation. This finding could be important for understanding the driving forces and folding pathways of the left-handed G-quadruplex structures. The extremely long residence time of the central ion could imply a lasting property of the *Z-G4* structure, making it an attractive target for drug design or as an oligonucleotide aptamer.

## Author contributions

ATP conceived the project; FRW, BB and PD performed NMR and CD experiments under the guidance of BH and ATP; AM and FR performed MS experiments under the guidance of VG; All authors discussed results and contributed to the writing of the manuscript.

## Conflicts of interest

There are no conflicts to declare.

## Supplementary Material

SC-012-D1SC00515D-s001
