# Supplementary material for: Unprecedented hour-long residence time of a cation in a left-handed G-quadruplex
Source: Chem Sci. 2021 Apr 26;12(20):7151–7. doi: 10.1039/d1sc00515d (PMC8153214; doi:10.1039/d1sc00515d)
Supplement: SC-012-D1SC00515D-s001 [file SC-012-D1SC00515D-s001.pdf]

Electronic Supplementary Material (ESI) for Chemical Science.
This journal is © The Royal Society of Chemistry 2021

Unprecedented hour-long residence time of a cation in the left-handed G-quadruplex

Fernaldo Richtia Winnerdy, Blaž Bakalar, Poulomi Das, Brahim Heddi, Adrien Marchand, Frédéric Rosu, Valérie Gabelica, Anh Tuân Phan*

SUPPORTING INFORMATION

Experimental procedures

Sample preparation for CD and NMR experiments

DNA samples were ordered from IDT with standard desalting. Sample purity, measured with ESI-MS, was >99%. The samples were folded in appropriate buffer to a final concentration 0.1-0.3 mM, annealed by heating to 100 °C and slowly cooled overnight to room temperature. Samples for CD spectroscopy were prepared by diluting the NMR sample in a buffer of the same composition to a final concentration of 2-8 µM. CD was immediately measured using a 1 cm path length quartz cuvette on a Jasco J-815 spectrometer. Molar elipticity of CD spectra was calculated using the DNA concentration derived from the sample absorbance at 260 nm and its extinction coefficient.

NMR experiments were performed on 600/800 MHz Bruker spectrometers equipped with cryoprobe, at 25°C with DNA concentrations 0.1–0.3 mM, unless otherwise stated. All spectra were processed and analyzed using TopSpin 2.1 (Bruker).

D_2O exchange NMR spectroscopy

Previously prepared NMR samples were flash frozen in liquid nitrogen and subsequently lyophilized. Just before the start of the experiment, an amount of D_2O was added that was equal to the previous sample volume and the sample was immediately measured on a NMR spectrometer.

Tracking of $^{15}NH_4^+$ using ^1H NMR spectroscopy

A sample of *Z-G4* was folded in either 100 mM $^{15}NH_4^+/K^+$ and subsequently dialyzed against H_2O for 10 minutes in order to remove most of the $^{15}NH_4^+/K^+$ ions in solution. It was quickly flash frozen in liquid nitrogen and lyophilized. Just before the start of the experiment, the sample was re-suspended in a buffer containing the opposite ion, either 100 mM $K^+/^{15}NH_4^+$ ions. The sample was immediately tracked with NMR where we interchangeably recorded both ^1H and ^{15}N filtered ^1H NMR spectra over 3 hours.

Tracking of dissociation of K^+ and NH_4^+ ion using using ^1H NMR spectroscopy

A sample of *Z-G4* was folded in either 100 mM $^{15}NH_4^+/K^+$ and subsequently dialyzed against H_2O for 10 minutes in order to remove most of the $^{15}NH_4^+/K^+$ ions in solution. It was quickly flash frozen in liquid nitrogen and lyophilized. Just before the start of the experiment, the sample was re-suspended in deionized H_2O.

Sample preparation for ESI-MS experiments

A sample of *Z-G4* was folded in 90 mM TMAA pH = 7, 10 mM KCl to a concentration of 0.1 mM, heated to 90 °, slowly cooled overnight and left for 7 days. Before measurements the sample was diluted 10x by either 100 mM TMAA pH = 7 or 100 mM NH_4OAc pH = 7. In both cases, after the 10x dilution, the effective concentration of K^+ in solution was 1 mM, while the sample concentration was 0.01 mM.

100 mM TMAA buffer was used when measuring the sample behavior in K^+ as TMAA does not coordinate between tetrads, due to its bulky nature, and is added to maintain ionic strength.

100 mM NH_4OAc buffer was used when tracking the conversion from K^+ to NH_4^+. The effective concentration of the NH_4^+ ion after the 10x dilution is 90 mM making the K^+/NH_4^+ ratio 1/90.

Samples were injected in the negative-ion ESI-MS with softest possible experimental tuning, that preserved the most NH_4^+ ion species. Samples were tracked through time by periodical injections and the recorded data was cleaned of non-specific adducts.

Electrospray ion mobility-mass spectrometry measurements

The experiments were carried out on an Agilent 6560 IMS-Q-TOF (Agilent Technologies, Santa Clara, CA), with its drift tube ion mobility cell operated in helium. The helium pressure in the drift tube was 3.89 ± 0.01 Torr, and the pressure in the trapping funnel is 3.63 ± 0.01 Torr. The pressure differential between the drift tube and the trapping funnel ensures only helium is present in the drift tube. Injection was in negative ion mode, using the standard electrospray source and a syringe pump at 4 µL/min. The acquisition software version was B.07.00 build 7.00.7008. The arrival time distributions were extracted from the entire isotopic distribution of each adduct, using IM-MS browser

Step-field experiments (five drift tube voltages for each samples) were performed to determine the CCS. The arrival time distributions (ATDs) for each charge state of the complexes were fitted with one gaussian peak using OriginPro 2016, to determine the arrival time t_A of the center of the peak. The arrival time t_A is related to ΔV (voltage difference between the entrance and the exit of the drift tube region) by:

$$t_A = \frac{L^2 T_0 p}{K_0 p_0 T} \cdot \left(\frac{1}{\Delta V}\right) + t_0 \qquad \text{(S1)}$$

t_0 is the time spent outside the drift tube region and before detection. A graph of t_A vs. $1/\Delta V$ provides K_0 from the slope and t_0 as the intercept. The drift tube length is L = 78.1 cm, the temperature is measured accurately by a thermocouple (here, T = 297 ± 1 K), and the pressure is measured by a capacitance gauge (p = 3.89 ± 0.01 Torr). The CCS is then determined using Equation (S2):

$$CCS = \frac{3ze}{16N_0} \cdot \sqrt{\frac{2\pi}{\mu k_B T}} \cdot \frac{1}{K_0} \qquad \text{(S2)}$$

The reconstruction of the experimental CCS distributions from the arrival time distributions at the lowest voltage is then performed using Equation (S3), where the factor *a* is determined from the t_A of the peak center at the lowest voltage and the *CCS* calculated from the regression described above, from the peak centers.

$$CCS = a \cdot \frac{z}{\sqrt{\mu}} \times t_A \tag{S3}$$

We checked with a method described elsewhere[1] whether diffusion outside the IMS would significantly contribute to the peak width, but we found that this contribution would contribute to only 1-2% of the peak width a the lowest voltage. Hence using Equation (S3) renders well the actual width of the CCS distribution.

Generation of gas-phase structures and calculation of theoretical collision cross sections

The gas-phase modeling was started from the published X-ray structure (PDB code 4U5M). Protons were added, and all but eight phosphate groups (arbitrarily chosen) were neutralized by protons, to attain a total charge state of 5- with 3 K^+ ions inside. We optimized the structure at the PM7 semi-empirical level[2] using Gaussian 16 rev. B.01[3]. Then, atom Centered Density Matrix Propagation molecular dynamics (ADMP, 1000 fs, 296 K) at the semi-empirical level (PM7) was also performed using Gaussian 16 rev. B01. The theoretical CCS values were calculated for a structure every 6 fs (every 30 steps), using the trajectory model (Mobcal[4], original parameters for helium, N and O parameterized as C, P and K parameterized as Si).

Isotope-exchange mass spectroscopy

The $^{41}K/^{39}K$ isotope exchange experiments were performed on a Bruker 7T SolariX XR ESI-Q-FTICRMS (Bruker, Bremen, Germany) with the ESI source operated in negative ion mode. The injection flow rate was 180 µL/h. All spectra were acquired in soft conditions.[5] The capillary exit voltage was -180 V, and skimmer 1 voltage was -5 V. The collision energy (entrance of the hexapole collision cell) was set to 1 V.

The stock solutions of the quadruplexes (50 µM strand concentration) were prepared in 100 mM trimethylammonium acetate (TMAA) with 5 mM KCl enriched to 96.5% ^{41}K. The solution was then diluted to 2.5 µM in quadruplex using a solution of 100 mM TMAA and 1 mM KCl (natural isotope ratio, i.e. 93.26% ^{39}K and 6.73% ^{41}K). The potassium exchange inside the quadruplex was probed as a function of time (25 min and 3 days). The end point was obtained upon annealing of the solutions at high temperature to disrupt the quadruplexes, cooling them down and leaving them at room temperature for 3 days prior to the mass analysis. Following dilution, the effective isotopic abundance of K^+ for the mixture were 78.8% in ^{39}K and 21.2% in ^{41}K.

Supplementary tables

Table S1. Sequences used in this work (5'-3')

Name	**Sequence**	**Number of nucleotides**	**ε_{260} (L mol^{-1} cm^{-1})**
Z-G4	TGGTGGTGGTGGTTGTGGTGGTGGTGTT	28	256100
2xBlock2Δ	GTGGTGGTGGTGTTGTGGTGGTGGTG	26	249400
ZG4-T4mod	TGGTTGGTGGTGGTTGTGGTGGTGGTGTT	29	272900
TG4T	TGGGGT	6	57800

Table S2. Buffers used in sample preparation

Buffer	Buffer composition
Potassium buffer	70 mM KCl, 20 mM KPi pH=6.9, 10% D_2O, 20 µM DSS
Ammonium buffer	100 mM ammonium acetate pH=6.9, 10% D_2O, 20 µM DSS
^{15}N Ammonium buffer	100 mM ^{15}N ammonium chloride, 20 mM deuterated Tris pH=6.9, 10% D_2O, 20 µM DSS
Sodium buffer	100 mM sodium chloride, 20 mM deuterated Tris pH=6.9, 10% D_2O, 20 µM DSS
Lithium buffer	100 mM Lithium chloride, 20 mM deuterated Tris pH=6.9, 10% D_2O, 20 µM DSS
Caesium buffer	100 mM caesium chloride, 20 mM deuterated Tris pH=6.9, 10% D_2O, 20 µM DSS

Table S3. summary of experimental and theoretical collision cross section values.

<table>
<tr><td></td><td colspan="2">Theoretical ($^{TM}CCS_{He}$ / Å²)</td><td>Experimental ($^{DT}CCS_{He}$ / Å²)</td></tr>
<tr><td rowspan="3">ZG4 with 3 K^+</td><td>X-ray (PDB: 4U5M)</td><td>955</td><td rowspan="3">891 ± 20[b]</td></tr>
<tr><td>PM7, optimized</td><td>875</td></tr>
<tr><td>PM7, ADMP molecular dynamics</td><td>877 ± 8[a]</td></tr>
<tr><td>ZG4 with 1 K^+/2 NH_4^+</td><td></td><td></td><td>898 ± 20[b]</td></tr>
<tr><td>ZG4 with 3 NH_4^+</td><td></td><td></td><td>894 ± 18[b,c]</td></tr>
</table>

[a] Standard deviation of the distribution.

[b] Full width at half-maximum of the reconstructed CCS distribution.

[c] The standard deviation on the value of the peak center in independent replicate experiments (N = 4) was 2.6 Å².

Supplementary figures

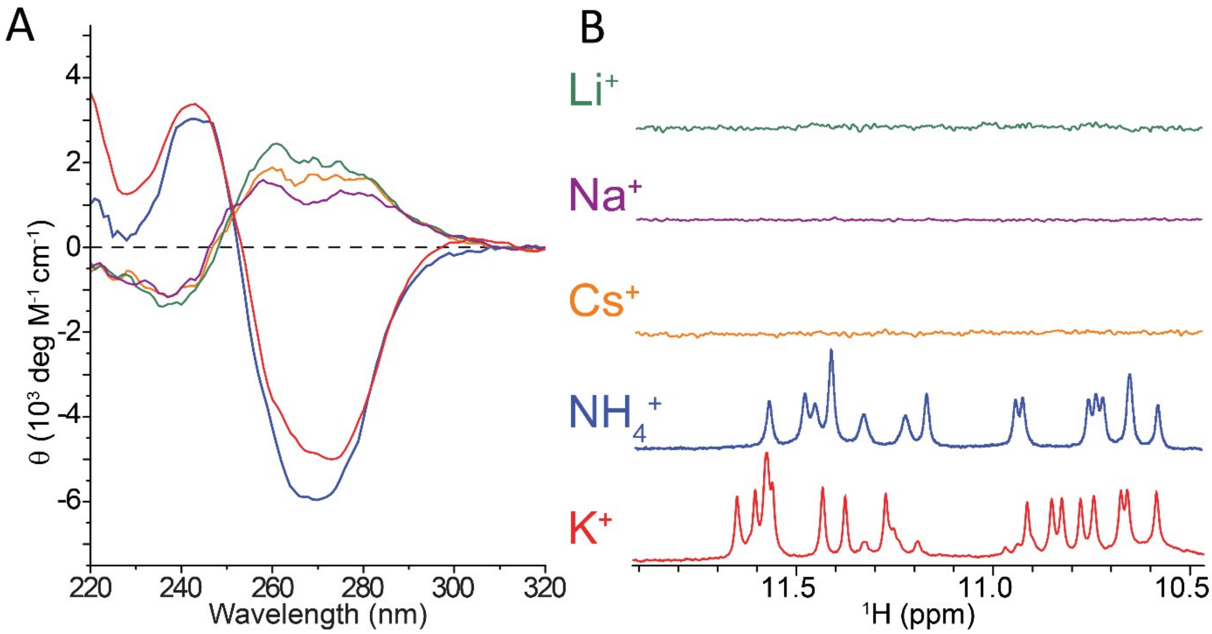

Figure S1. CD (A) and NMR (B) spectra of *Z-G4* folded in buffers with different cations.

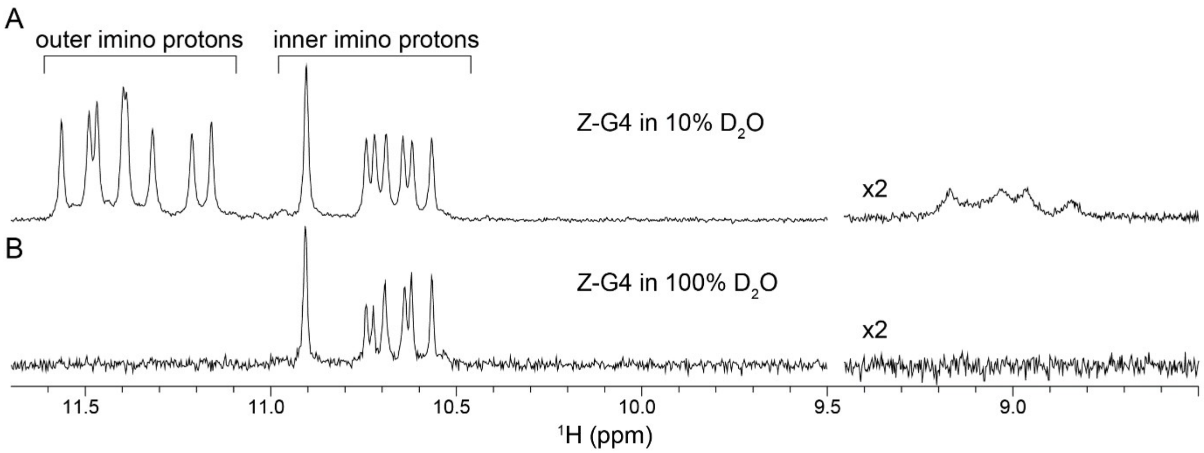

Figure S2. Imino and amino ^{1}H NMR spectra of *Z-G4* in NH_4^+ solution (A) before and (B) two weeks after D_2O exchange experiment. Only the outer G-tetrad imino protons were exchanged after two weeks, identifying the protected inner G-tetrad imino protons.

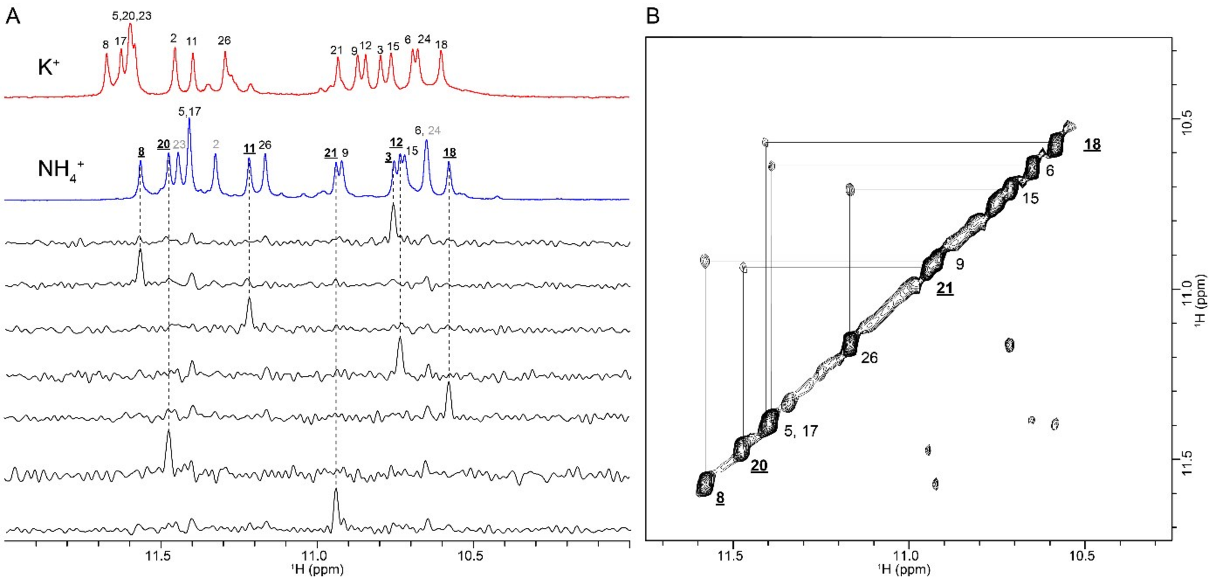

Figure S3. Imino peak assignment of *Z-G4* in NH_4^+. Underlined & Bold imino peaks (7) are assigned directly using ^{15}N labeled guanines, bold & asterisk imino peaks (6) are assigned using NOESY crosspeaks, while gray imino protons (3) are assumed based on similiarites with the 1D spectrum of *Z-G4* in K^+.

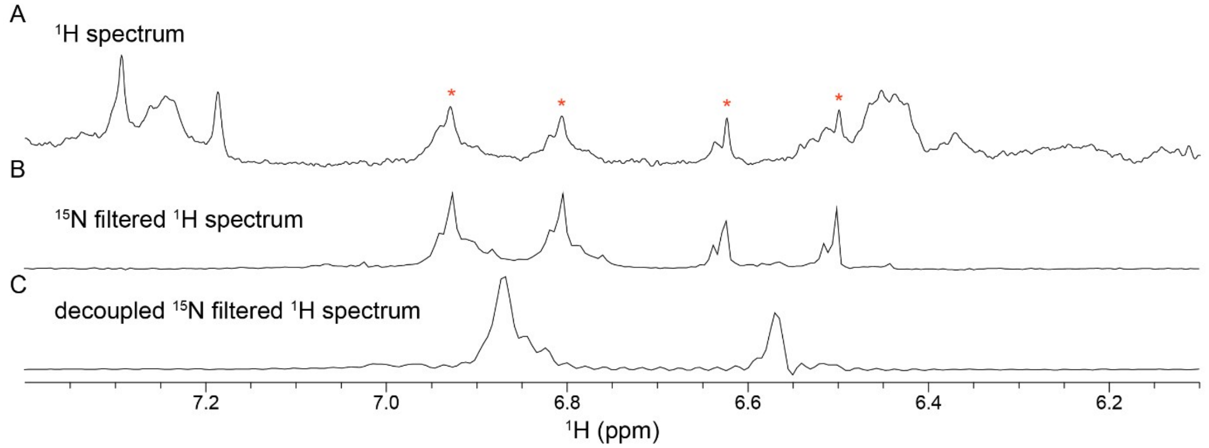

Figure S4. NMR spectra of *Z-G4* folded in $^{15}NH_4^+$. (A) ^{1}H NMR spectrum, (B) ^{15}N filtered NMR spectrum and (C) ^{15}N filtered proton-decoupled NMR spectrum. All spectra were recorded at 298 K.

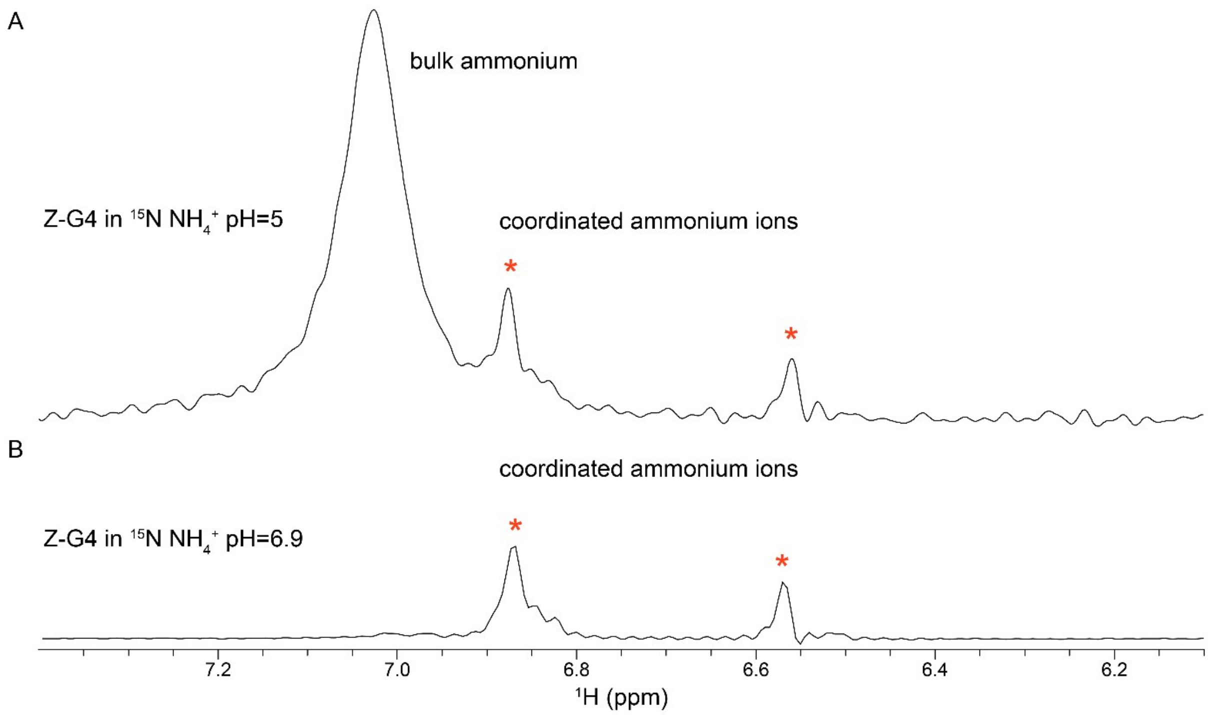

Figure S5. Proton-decoupled ^{15}N filtered NMR spectra of *Z-G4* folded in $^{15}NH_4^+$ buffer with either (A) pH=5 or (B) pH=6.9. Peaks of coordinated ammonium ions are labeled with red asterisk. Spectra were recorded at 298 K.

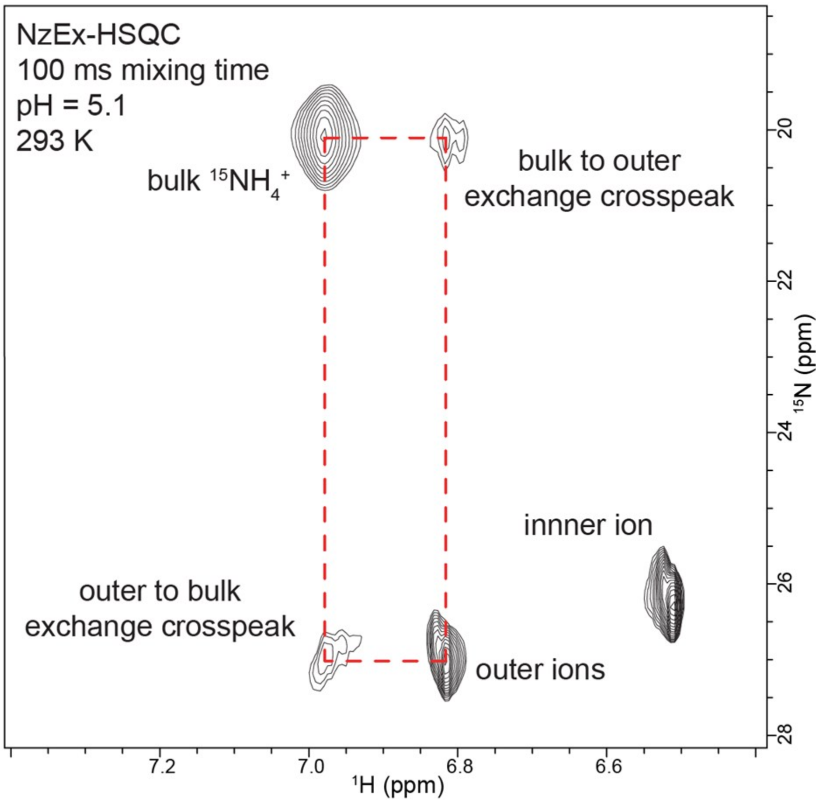

Figure S6. 2D NzEx-HSQC [6] spectrum recorded with a 100 ms mixing time at pH = 5.1 and 293 K. Bulk, outer and inner $^{15}NH_4^+$ ion peaks are appropriately annotated. The cross-peaks between the outer and bulk ions are shown, with a dashed red line connecting them.

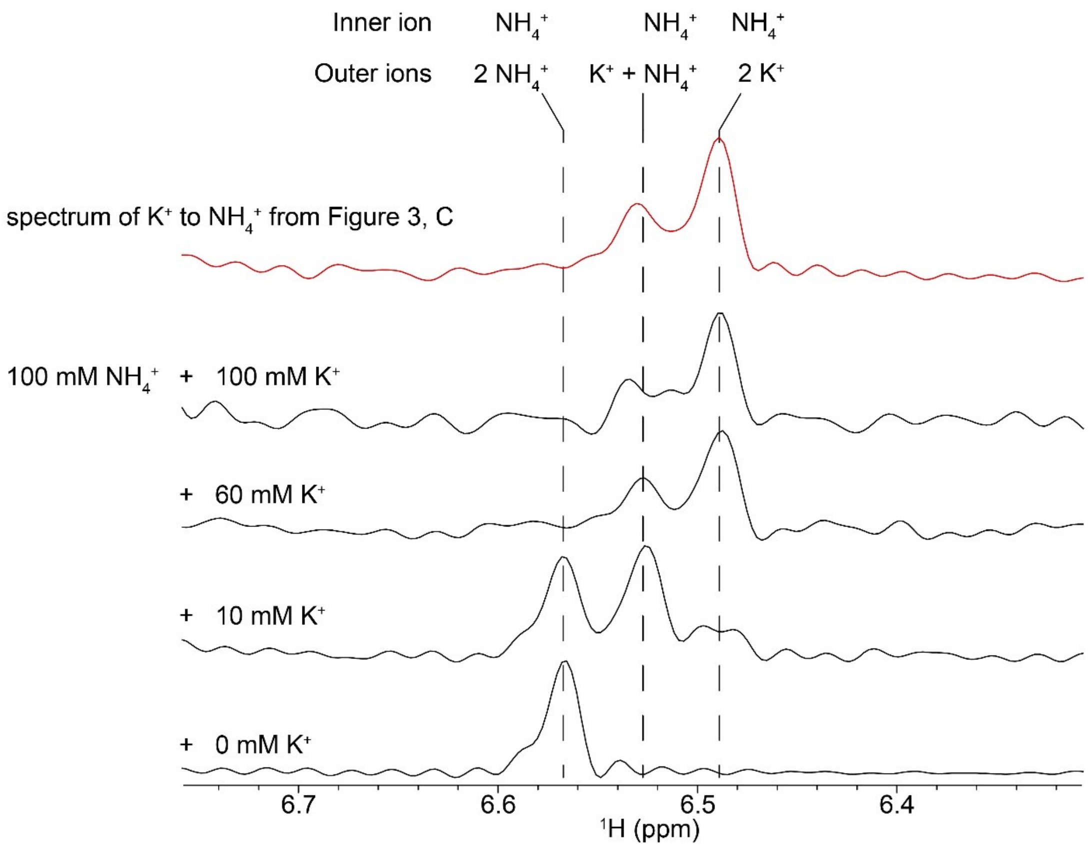

Figure S7. Comparison of inner $^{15}NH_4^+$ ion chemical shifts in proton-decoupled ^{15}N filtered NMR spectra, with varying concentration of added K^+ ions. The spectrum from Figure 3C is shown in red, vertical dashed lines represent different species, with the coordinated ions listed at the top.

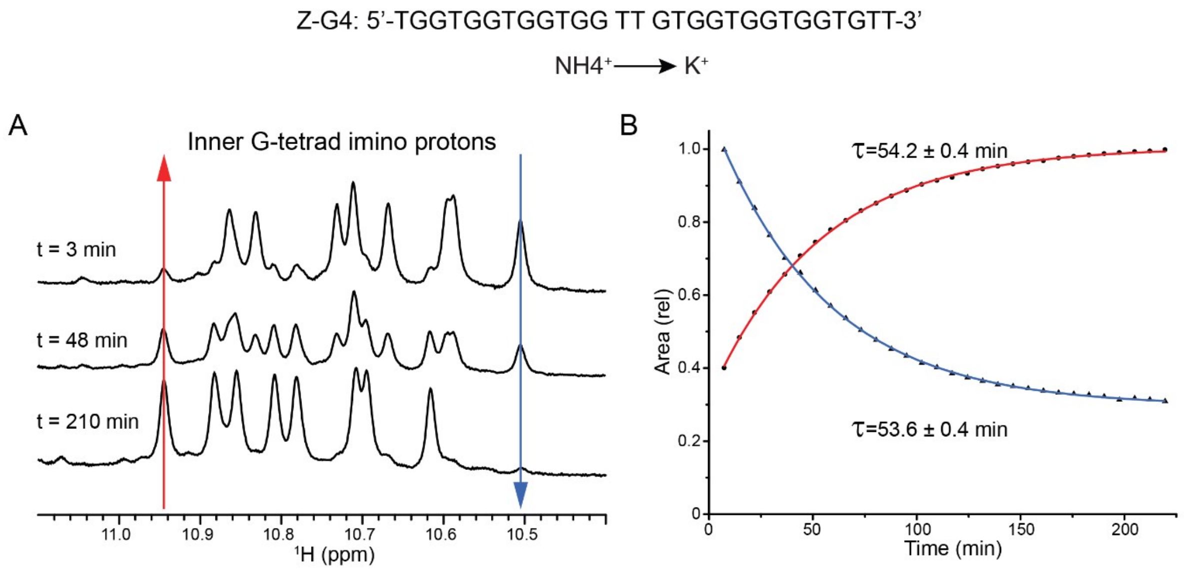

Figure S8. Tracking of *Z-G4* inner imino proton peak appearance and disappearance during the ion exchange process of NH_4^+ to K^+ with ^1H NMR. (A) Inner G-tetrad ^1H NMR imino proton peak region. Three spectra are shown representing different time points during the experiment. The red arrow shows the appearance of the inner G-tetrad imino proton peak with time (species with K^+) while the blue arrow shows the disappearance of the inner G-tetrad imino proton peak with time (species with NH_4^+). All growing imino proton peaks correspond to K^+-containing *Z-G4*, while all diminishing imino proton peaks correspond to NH_4^+-containing *Z-G4* (B) Graphs of the corresponding peak areas versus time.

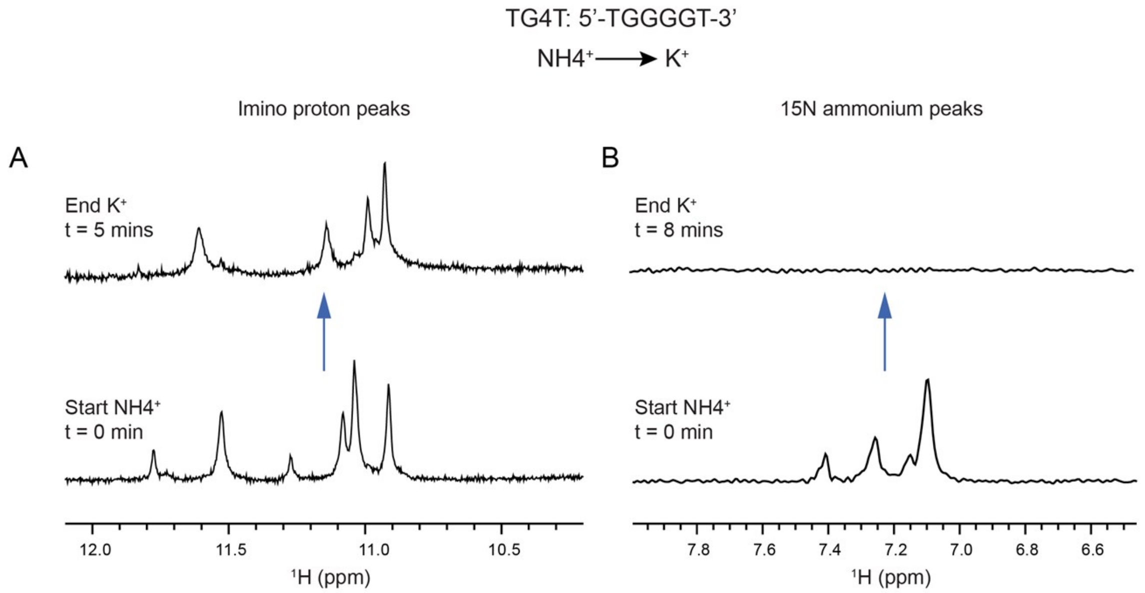

Figure S9. (A) Fast exchange of *TG4T* imino proton peaks observed with ^{1}H NMR in $^{15}NH_4^+$ to K^+ ion exchange experiment. The imino proton peaks corresponding to G4 with $^{15}NH_4^+$ quickly disappeared, and the ones corresponding to G4 with K^+ quickly appeared. (B) The same process observed with proton-decoupled ^{15}N-filtered ^{1}H NMR. The $^{15}NH_4^+$ peaks quickly disappeared upon addition of K^+, indicating fast-exchange regime.

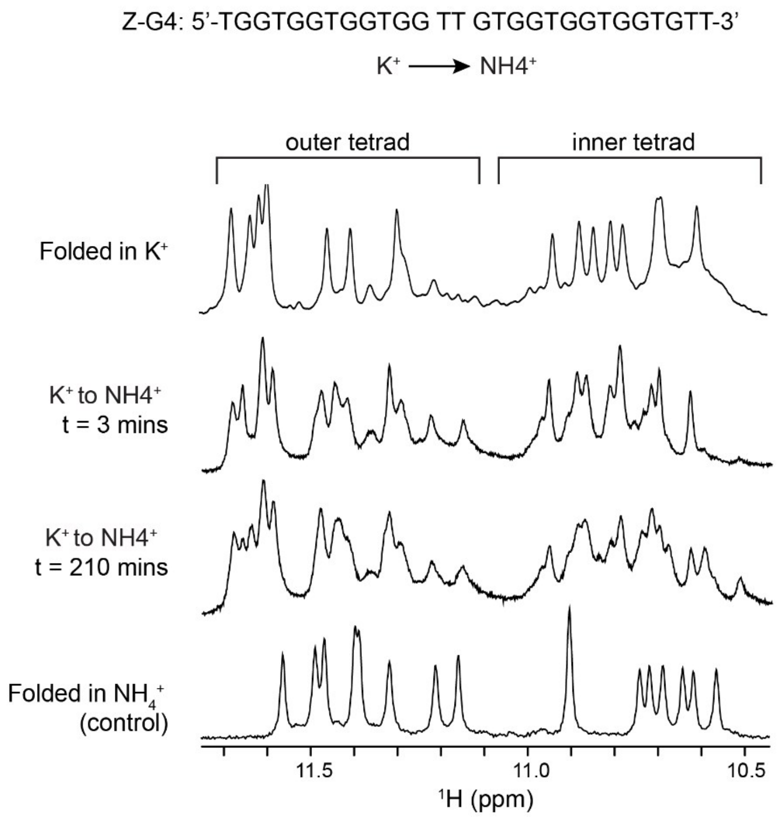

Figure S10. Tracking of imino proton peaks appearance and disappearance with ^{1}H NMR. The top and bottom spectra are of *Z-G4* folded in K^+ and $^{15}NH_4^+$ ion respectively. The middle two spectra are from the start (t = 3 mins) and end (t = 210 mins) of the $K^+ \rightarrow {}^{15}NH_4^+$ experiment.

ZG4: 5'-TGGTGGTGGTGG TT GTGGTGGTGGTGTT-3'

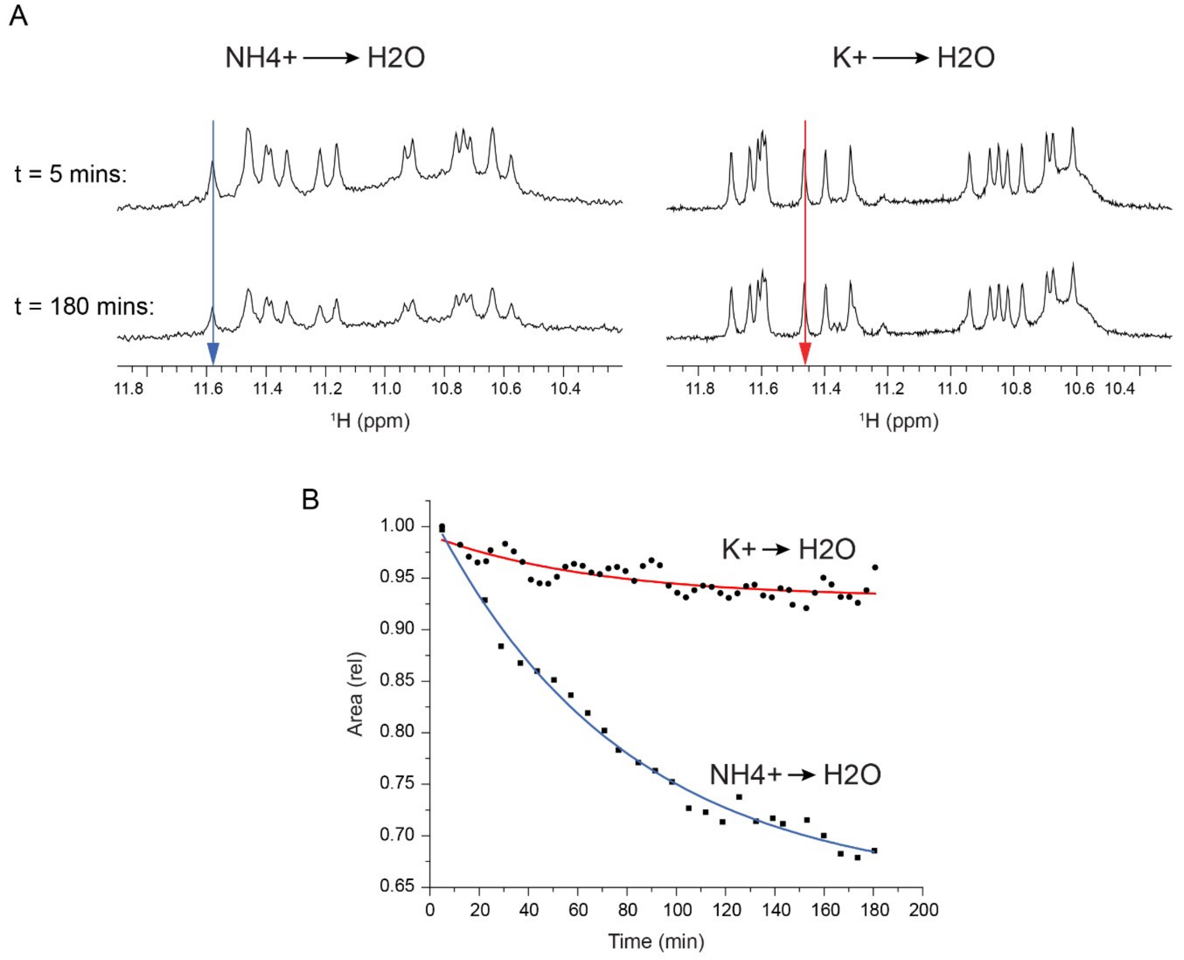

Figure S11. (A) Tracking of imino proton peaks with ^{1}H NMR when *Z-G4* folded in K^+ and NH_4^+ ions is dissociated in H_2O. (B) Plot of corresponding imino proton peak areas versus time.

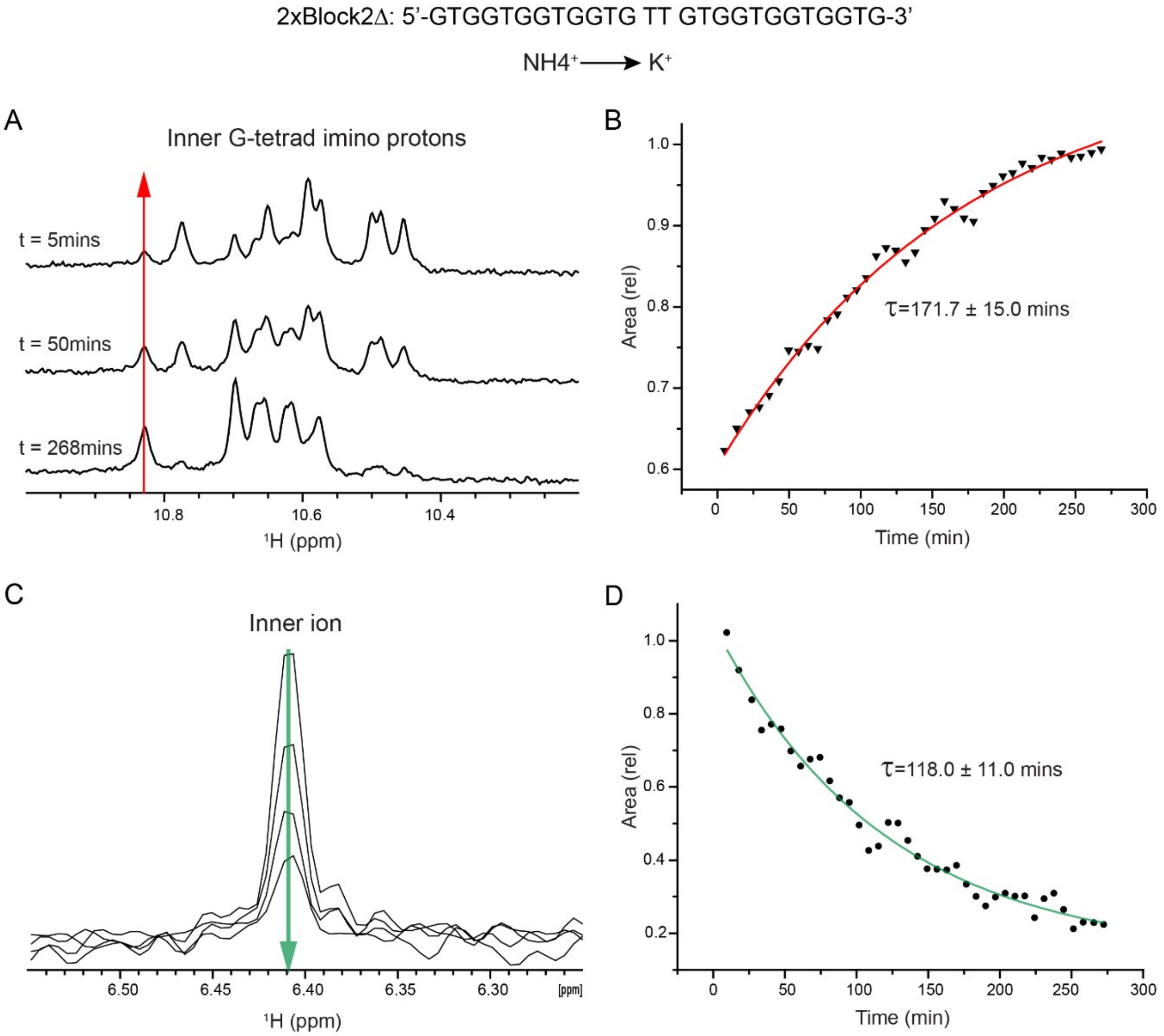

Figure S12. Tracking of peak appearance and disappearance of *2xBlock2Δ* during the ion exchange process of NH_4^+ to K^+ with ^1H NMR. (A) Inner G-tetrad ^1H NMR imino proton peak region. Three spectra are shown representing different time points during the experiment. The red arrow shows the appearance of the inner G-tetrad imino proton peak with time which is plotted on the graph (B). (C) Tracking of the inner ammonium peak disappearance as it is exchanged with the bulk K^+ with proton-decoupled ^{15}N-filtered ^1H NMR which is plotted in (D).

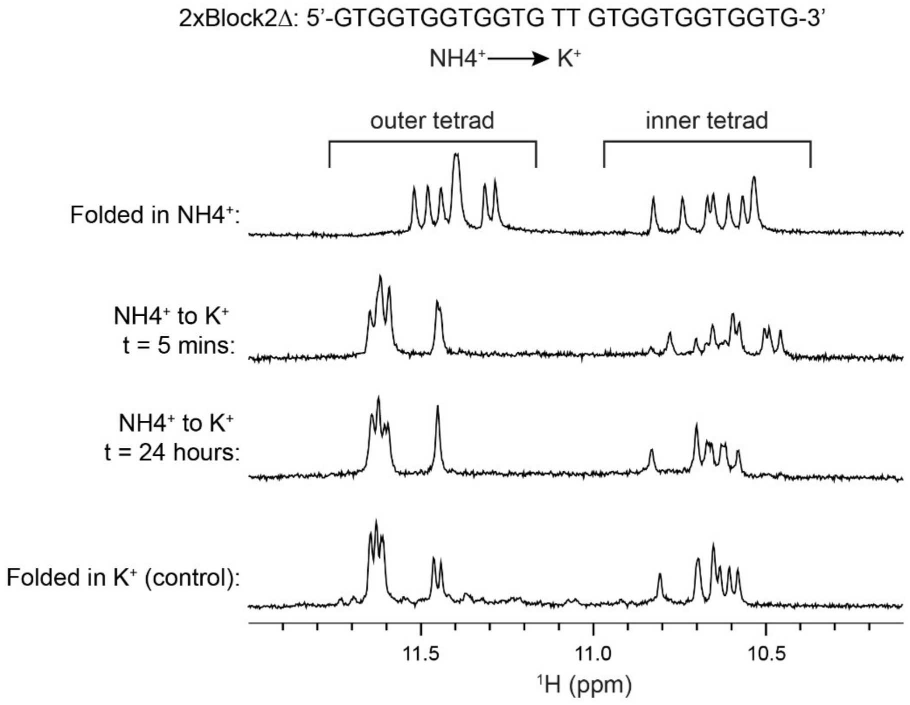

Figure S13. Tracking of imino proton peaks appearance and disappearance of *2xBlock2Δ* with ^{1}H NMR. The top and bottom spectra are of *2xBlock2Δ* folded in $^{15}NH_4^+$ and K^+ ion respectively. The middle two spectra are from the start (t = 5 mins) and end (t = 24 hours) of the $^{15}NH_4^+ \rightarrow K^+$ experiment.

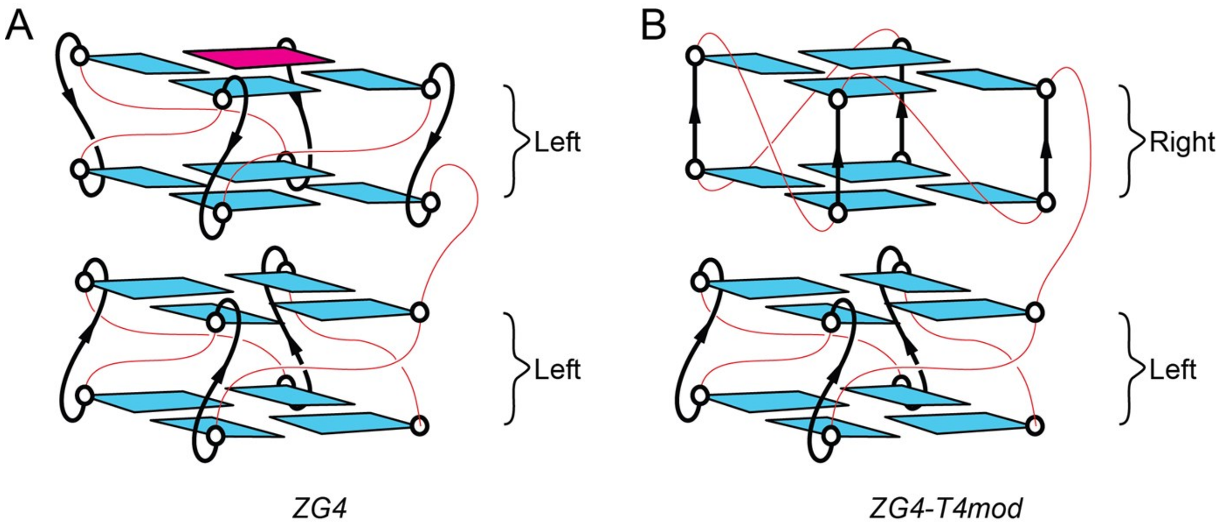

Figure S14. Schematics of (A) a left-handed G4 topology (*Z-G4*) and (B) a right-left-hybrid G4 topology (*ZG4-T4mod*). The stacking between two blocks for both structures are homologous, in a 5’-5’, opposite polarity mode. *Syn*- and *anti*-guanine are colored in magenta and cyan respectively.

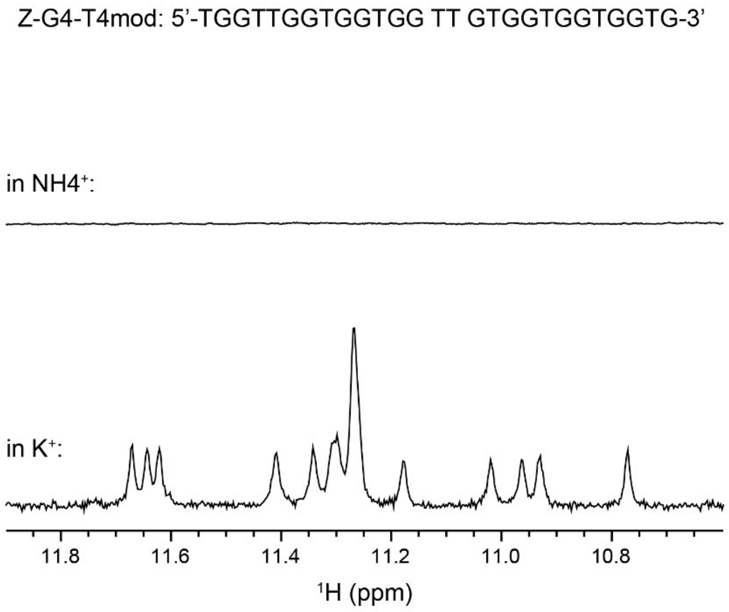

Figure S15. The imino proton region of ^{1}H NMR spectra of *ZG4-T4mod* folded in K+ and NH4+. *ZG4-T4mod* adopts right-left G4 conformation in K+ and is unable to fold into any structure in $NH4^+$.

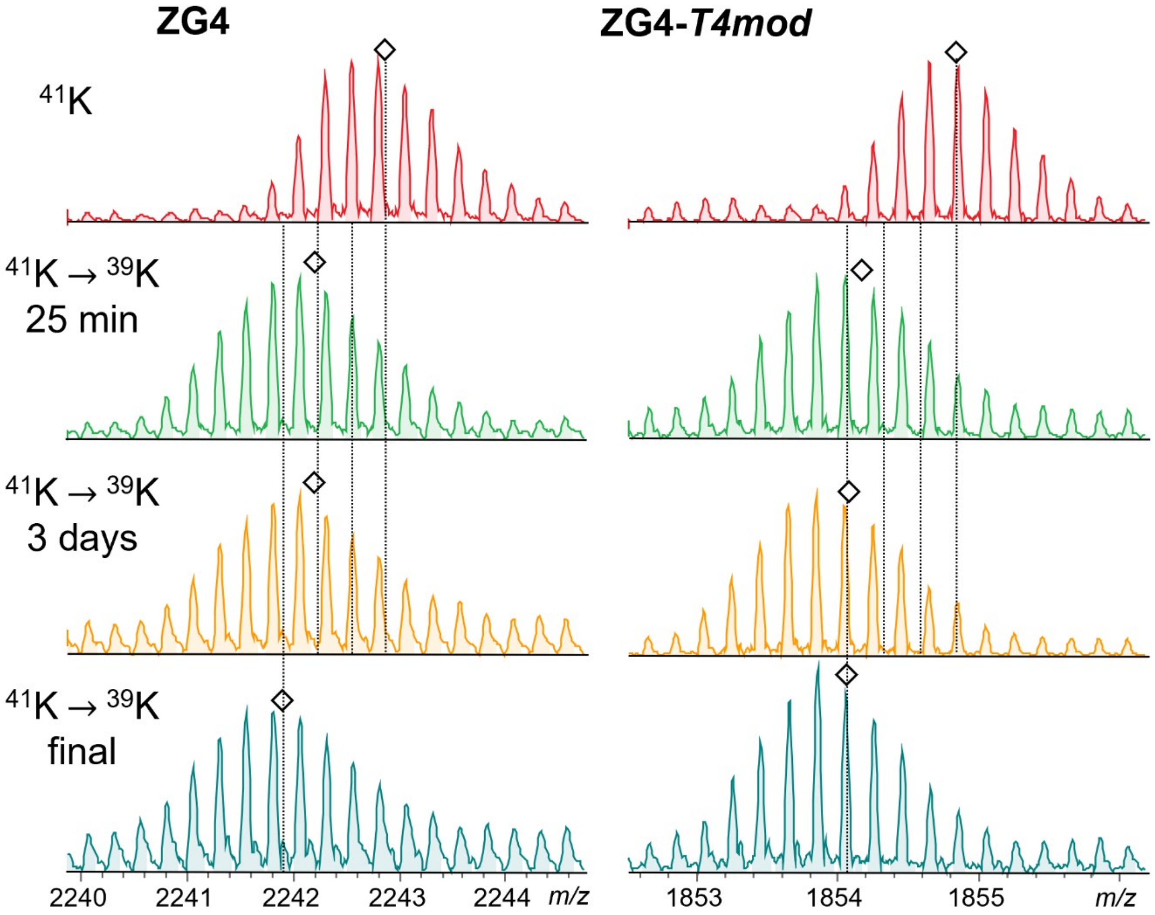

Figure S16. Mass spectroscopy kinetics experiments for *Z-G4* (left) and *ZG4-T4mod* (right), showing the istope cation exchange from ^{41}K to ^{39}K ions. The diamonds indicate the centroid of each isotopic distribution. The starting point (top spectrum) was recorded by dilution in ^{41}KCl. The end point (bottom spectrum) is recorded after annealing of the final solution in ^{39}KCl. The mass spectra after 25 minutes and 3 days of exchange are shown in the middle. The guidelines indicate the average centroid predicted for 0, 1, 2 and 3 cations exchanged. *Z-G4* exchanges 2 K^+ ions faster than 25 mins, while the last K^+ is still preserved after 3 days. The *ZG4-T4mod* exchanges nearly all three cations faster than 25 mins.

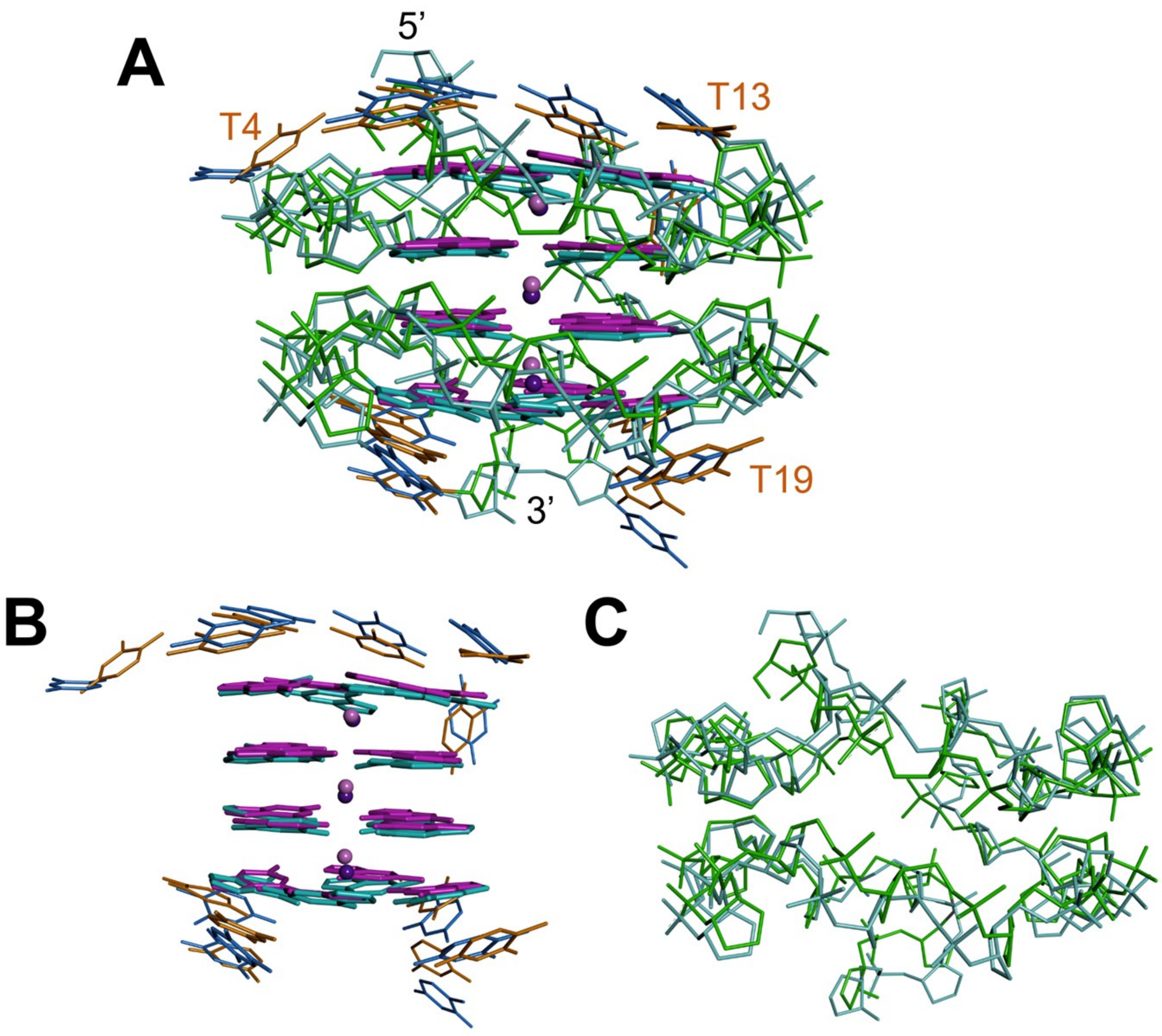

Figure S17. (A) Superimposition of the PM7 optimized structure (guanines in cyan, backbone in green, thymines in brown) and the X-ray structure (PDB: 4U5M) (guanines in pink, backbone in light blue and thymines in dark blue). The RMSD between the two structures is 1.43 Å^2, the main contribution coming from rearrangment of thymines (B) and backbone (C).

References

1. A. Marchand, S. Livet, F. Rosu and V. Gabelica, *Analytical chemistry*, 2017, **89**, 12674-12681.
2. J. J. Stewart, *Journal of molecular modeling*, 2013, **19**, 1-32.
3. M. Frisch, G. Trucks, H. Schlegel, G. Scuseria, M. Robb, J. Cheeseman, G. Scalmani, V. Barone, G. Petersson and H. Nakatsuji, *There is no corresponding record for this reference*.
4. M. Mesleh, J. Hunter, A. Shvartsburg, G. C. Schatz and M. Jarrold, *The Journal of Physical Chemistry*, 1996, **100**, 16082-16086.
5. F. Balthasart, J. Plavec and V. Gabelica, *J Am Soc Mass Spectrom*, 2013, **24**, 1-8.
6. N. V. Hud, P. Schultze, V. Sklenar and J. Feigon, *J Mol Biol*, 1999, **285**, 233-243.
